# Association between herpes simplex virus 1 exposure and the risk of depression in UK Biobank

**DOI:** 10.1002/ctm2.108

**Published:** 2020-06-20

**Authors:** Jing Ye, Yan Wen, Xiaomeng Chu, Ping Li, Bolun Cheng, Shiqiang Cheng, Li Liu, Lu Zhang, Mei Ma, Xin Qi, Chujun Liang, Om Prakash Kafle, Yumeng Jia, Cuiyan Wu, Sen Wang, Xi Wang, Yujie Ning, Feng Zhang

**Affiliations:** ^1^ Key Laboratory of Trace Elements and Endemic Diseases of National Health and Family Planning Commission School of Public Health, Health Science Center Xi'an Jiaotong University Xi'an China

**Keywords:** depression, gene‐environment interaction, herpes simplex virus (HSV)

## Abstract

**Background:**

Herpes simplex virus‐1 (HSV‐1) infection is reported to be associated with depression. But limited efforts were made to investigate the relationship between HSV‐1 infection and the risk of depression, especially from the genetic perspective.

**Methods:**

In UK Biobank cohort, linear and logistic regression analyses were first performed to test the association of HSV‐1 seropositivity/antibody with depression, including depression status (N = 2951) and Patient Health Questionnaire (PHQ) score (N = 2839). Using individual genotypic and phenotypic data from the UK Biobank, genome‐wide environmental interaction study (GWEIS) was then conducted by PLINK2.0 to evaluate gene × HSV‐1 interacting effect on the risk of depression. Finally, gene set enrichment analysis was conducted to identify the biological pathways involved in the observed gene × HSV‐1 interaction for depression.

**Result:**

In UK Biobank cohort, significant associations were observed between depression status and HSV‐1 (odds ratio [OR] = 1.09; 95% confidence interval [CI], 1.02‐1.16; *P* = 2.40 × 10^−2^ for HSV‐1 antibody and OR = 1.28; 95% CI, 1.12‐1.47, *P* = 2.59 × 10^−3^ for HSV‐1 seropositivity). GWEIS revealed four significant gene × HSV‐1 interaction signals for PHQ score (all *P *< 5.0 × 10^−8^) and the leading loci was SULF2 (rs6094791, *P = *8.60 × 10^−9^). Pathway analyses identified 21 pathways for PHQ score and 19 for depression status, including multiple neural development‐ and immune‐related ones, such as KEGG_NEUROACTIVE_LIGAND_RECEPTOR_INTERACTION (false discovery rate [FDR] = 3.18 × 10^−2^) for depression and LU_AGING_BRAIN_UP (FDR = 4.21 × 10^−2^) for PHQ score.

**Conclusion:**

Our results suggested that HSV‐1 was associated with the risk of depression, which was modulated by the several genes that were related to the nerve development or immune function.

## 1 INTRODUCTION

Depression is a mental disorder characterized by persistent sadness and a lack of interest or pleasure. According to the estimation of World Health Organization, there are 322 million people or 4.4% of the global population affected by depression. It is found that that the leading cause of disease‐related disability is depression,^1^ which is also related with higher cancer incidence and cancer‐specific mortality.^2^ Because treatment of depression can only alleviate rather than cure the condition, the burden of depression outcome continues to grow, especially suicide. Therefore, the identification and prevention of the high‐risk group of depression is an effective and key measure.

Previous studies have demonstrated the important roles of genetic factors in the development of depression. Twin and family studies suggested that approximately 30‐40% of unipolar depression could be explained by genetic effects.^3^ A meta‐analysis of 807 553 individuals from three largest genome‐wide depression association studies identified 102 independent variants and 269 genes associated with depression.^4^

It is clear from previous studies that a large proportion of phenotypic variance of human complex diseases and traits cannot be explained by genetic factors, which might come from environmental or genetic effects mediated by environmental exposure. Genome‐wide environment interaction studies (GWEISs) are helpful for discovering new genetic risk variants, and understanding the potential biological mechanisms.^17^ For instance, Rivera et al revealed 53 and 34 additive interactions of single nucleotide polymorphism(SNP) and smoking in Löfgren's syndrome (LS) and non‐LS, respectively, but no association was identified from assessing the effect of smoking on sarcoidosis without genetic information.^5^

The risks of depression depend on individual genetics, environment, and the complex interaction between them. Recently, abnormal immune response and increased inflammation have been suggested to be a risk factor for psychiatric disorders, especially depression.^6^, ^7^, ^8^ Herpes simplex virus 1 (HSV‐1) is able to promote beta‐amyloid deposition and tau phosphorylation. It has been proved to promote cognitive deficits relevant to bipolar disorder^9^ and schizophrenia.^10^ Waubant et al illustrated that in multiple sclerosis, HSV‐1 seropositivity can lead to increased risk among those patients without the DRB1*15 allele through gene‐environment interactions study.^11^ However, limited efforts have been paid to explore the modulating effect of SNP in the association between HSV‐1 and depression.

Utilizing the UK Biobank cohort, we first tested the association between HSV‐1 infection and depression. GWEIS was then performed to identify gene × HSV interacting effect on the risk of depression. Finally, gene set enrichment analysis was used to detect the biological pathways contributing to the association observed between HSV‐1 and depression.

## 2 METHODS

### 2.1 UK Biobank cohort

The analysis data of study individuals were extracted from the UK Biobank health resource (http://www.ukbiobank.ac.uk/about-biobank-uk/). The UK Biobank has performed a large prospective population‐based cohort study, comprising health‐linked information, hospital record, and genetic data of 502 656 participants aged 40‐69 years in 2006 and 2010. All participants agreed to use their anonymous data and samples for any health‐related research, to reconnect for further sub‐studies.^12^ The present study has been approved by UK Biobank and gets the access to participants’ health‐related records, including HSV‐1 antibody, HSV‐1 serum reaction, and self‐reported depression status.

### 2.2 UK Biobank phenotypes of HSV‐1 and depression

A total of 9691 participants were assayed at German Cancer Research Centre (DKFZ) using multiple serology.^13^ Multiple serology^13^ was performed at the serum dilution 1:1000.^14^ HSV‐1 antibody (UK Biobank code: 23000) and HSV‐1 seropositivity (UK Biobank code: 23050) were used to represent HSV‐1 infection in this study. HSV‐1 seropositivity is a binary variable and HSV‐1 antibody is a continuous variable. In order to classify the subjects as accurate as possible, strict criterion based on Patient Health Questionnaire (PHQ‐9) and composite international diagnostic interview short‐form (CIDI‐SF)^15^, ^16^ were used for inclusion and exclusion. PHQ‐9 is a classification algorithm with a total score (0‐27) for screening and measuring depression severity. It mainly focuses on nine depressive symptoms (full details in the Supporting Information). Based on the previous study,^17^ the continuous variables (HSV‐1 antibody and PHQ score) were subtracted from their mean, and divided by their standard deviation.

### 2.3 Statistical analysis

Logistic regression analysis was applied for evaluating the association between HSV‐1 and depression in UK Biobank cohort. Linear regression analysis was applied for evaluating the association between HSV‐1 and PHQ score. HSV‐1 antibody and HSV‐1 seropositivity were used as instrument variable in regression analysis. PHQ score and depression were used as outcome variables. For each regression analysis, sex, age, 10 principle components of population structure, smoking, alcohol use, and Townsend deprivation index were used as covariates (the definition of smoking, alcohol use, and Townsend deprivation index is in the Supporting Information). All analyses were conducted by R 3.5.3.

### 2.4 Sensitivity analysis

We derived PHQ score into category variable and performed the same analysis. The quarters of PHQ score were used to define the control and case group, using a logistic regression model to estimate the association between PHQ score and HSV‐1. Meanwhile, in order to estimate the co‐relationship of self‐reported depression and PHQ score, the spearman correlation approach and a logistic regression model were used, in which self‐reported depression was used as outcome variable and PHQ score was used as instrumental variable. All analyses were conducted by R 3.5.3.

**Graphical Headlights**

1. Our results showed the association between herpes simplex virus‐1 (HSV‐1) and depression.
2. Genome‐wide environmental interaction study (GWEIS) results indicated that several genes that related to the nerve development or immune function could modulate the association between depression and HSV‐1.
3. Pathway analyses identified 21 pathways for PHQ score and 19 for depression status, including multiple neural development and immune related ones.

### 2.5 UK Biobank genotyping, imputation, and quality control

A total of 488 377 participants were genotyped by either the UK Bileve array or the UK Biobank axiom array. Details of the array design, genotyping, and quality control procedures have been described elsewhere.^18^ These genotype results were subsequently used to make imputation based on Haplotype Reference Consortium (HRC) reference panel^19^ (version 1.1) and UK10K and 1000 Genomes project reference panels.^20^ Participants filtering criteria are as follows, with inconsistencies between self‐reported gender and genetic gender, without imputation data, and without ethic consents. Additionally, individuals were restricted to the “White British” group based on self‐reported ethnicity (UK Biobank field ID: 21000). UK used an estimator implemented in the KING software to remove genetically related individuals.^18^

### 2.6 GWEIS analysis

The generalized linear regression model of PLINK2.02^1^ was used to estimate the gene × HSV‐1 interaction effects on the risk of depression, using age, gender, and the first 10 European‐specific principal components as covariates. Briefly, gene‐environment interactions result from individuals responding differently to environmental stimuli based on their genotype.^22^ Based on the previous study,^5^, ^23^ the genetic additive (ADD) and dominance (DOM) models of PLINK2.0 were used. Additional quality control filters were used to select high‐quality SNPs: the SNPs with low call rates (<0.90), low Hardy‐Weinberg equilibrium exact test *P*‐values (<.001), or low minor allele frequencies (<0.01) were excluded.^21^ Significant and suggestive interactions were identified at *P* < 5.0 × 10^–8^ and *P* < 5 × 10^–7^, respectively. Visualization of all results was done using R software. Circular Manhattan plots of GWEIS results were generated using the “CMplot” R script (https://github.com/YinLiLin/R-CMplot). Subsequently, we conducted subgroup analysis through stratification according to significant SNP status. We selected the participants having the risk allele as one group and the participants without risk allele as another group. Then we used the linear regression and logistic regression model to estimate the associations between depression and HSV‐1 in each subgroup.

### 2.7 Pathway enrichment analyses

The GWEIS results of PHQ score and depression were further subjected to pathway enrichment analysis. Pathway enrichment analysis is a powerful approach to interpret genome‐wide studies data for complex diseases, by jointly considering multiple variants in interacting or functionally related genes.^24^ It was conducted using the modified gene‐set enrichment analysis (GSEA) algorithm.^24^ The modified GSEA algorithm calculates the maximum statistics of all SNPs mapping to known genes, and then uses similar running sum statistics such as Kolmogorov‐Smirnov to combine the effects of genes in the same path to calculate the maximum of each gene statistics, which increases the chances of identifying genetic variations that have little impact on disease risk.^24^ Four public pathway databases were collected to build pathway database, including KEGG, BioCarta, Ambion GeneAssist Gene Pathway Atlas, and Gene Ontology (GO). A total of 20 000 permutations were conducted to calculate the false discovery rate (FDR) of each analyzed pathway. Significant pathways were identified at FDR < 0.05. Furthermore, we obtained the significant pathways of depression and PHQ score to explore the distribution of pathways between the two groups through “Venn Diagram” package of R.

## 3 RESULT

### 3.1 Descriptive characteristics of study samples

A total of 2839 participants completed the PHQ with 58.6% women, and the mean (standard deviation [SD]) age was 55.9 (7.9) years old. A total of 2951 participants answered the depression‐related questions, and 1464 were classified into depression group with 58.9% women, and the mean (SD) age was 56.2 (7.8) years old. The quarters of PHQ score were 0 and 4, so 769 participants with PHQ score ≥ 4 were selected as case group and 922 participants with PHQ score ≤ 0 were selected as control group. And 57.7% of those were women, and the mean (SD) age was 55.6 (8.0) years old (Table 1).

**Table 1:** Basic characteristics of study sample from UK Biobank

		HSV‐1 antibody/seropositivity
Depression	Case/Control	1464/1487
	Sex (Female)	1737 (58.9%)
	Age (SD)	56.2 (7.8)
PHQ score (Continue variable)	Sample	2839
	Sex (Female)	1664 (58.6%)
	Age (SD)	55.9 (7.9)
PHQ score (Category variable)	Case/Control	769/922
	Sex (Female)	976 (57.7%)
	Age (SD)	55.6 (8.0)

*Note*. Age was described as Mean (standard deviation). The PHQ score is called the patient health questionnaire score and is used to describe the depression status of the participants.

Abbreviation: PHQ, Patient Health Questionnaire.

### 3.2 Association between depression and HSV‐1 in UK Biobank cohort

In UK Biobank cohort, significant associations were observed between depression and HSV‐1 antibody (odds ratio [OR] = 1.09; 95% confidence interval [CI], 1.02‐1.16; *P* = 2.40 × 10^−2^), and depression and HSV‐1 seropositivity (OR = 1.28; 95% CI, 1.12‐1.47; *P* = 2.59 × 10^−3^). The associations were also observed between PHQ score and HSV‐1 antibody (*B* = .02, standard error(SE) = 0.02, T‐test(*T)* = 1.19, *P* = 2.33 × 10^−1^), and PHQ score and HSV‐1 seropositivity (*B* = .01, SE = 0.04, *T* = 0.35, *P* = 7.26 × 10^−1^) (Table 2).

**Table 2:** Association among depression, PHQ score, and HSV‐1

Outcome	Instrument	*B*	SE	*T*	*P*‐value	OR (95% CI)
Depression	HSV‐1 antibody	.09	0.04	2.26	.0240	1.09 (1.02‐1.16)
	HSV‐1 seropositivity	.25	0.08	3.01	.0026	1.28 (1.12‐1.47)
PHQ score (Continue variable)	HSV‐1 antibody	.02	0.02	1.19	.2329	–
	HSV‐1 seropositivity	.01	0.04	0.35	.7257	–
PHQ score (Category variable)	HSV‐1 antibody	.09	0.05	1.68	.0939	1.09 (1.00‐1.19)
	HSV‐1 seropositivity	.11	0.11	1.02	.3074	1.12 (0.94‐1.33)

*Note*. Logistic regression was used to test the association between depression and HSV‐1. Linear regression was used to test the association between PHQ score and HSV‐1.

Abbreviations: CI, confidence interval; OR, odd ratios; PHQ, Patient Health Questionnaire;SE, standard error;T, ‐test.

### 3.3 Sensitivity analysis

We found the similar association between PHQ score and HSV‐1 after deriving PHQ score into category variable. The quarters of PHQ score were 0 and 4 in UK Biobank. We did not observe the significant associations between PHQ score and HSV‐1 antibody (OR = 1.09; 95% CI, 1.00‐1.19; *P* = 9.39 × 10^−2^), and PHQ score and HSV‐1 seropositivity (OR = 1.12; 95% CI, = 0.94‐1.33; *P* = 3.07 × 10^−1^) (Table 2). These results of novel analyses of PHQ‐driven categorical variable were same as that of previous continuous variables of PHQ score. The correlation between self‐reported depression and PHQ score was significant (*R* = .40, *P* < 1.00 × 10^−8^ for spearman correlation and *B* = .44, *P* < 1.00 × 10^−8^ for logistic regression model).

### 3.4 GWEIS results

**Figure 1:**
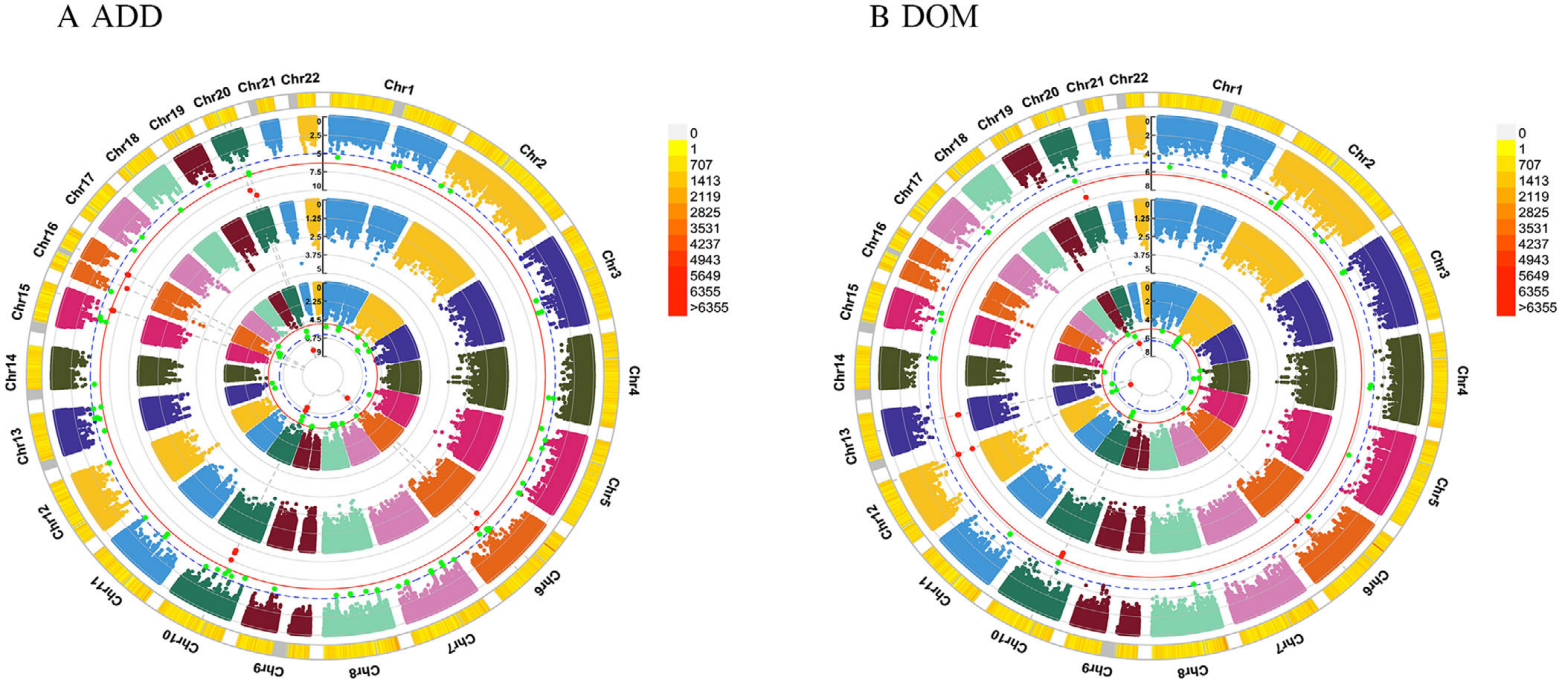
**Genomic regions interacting with HSV‐1 for PHQ score** *Note*. From the center, the first circos depicts the –log_10_ *P*‐values of each variant due to double exposure, that is, the effect of both SNP allele and HSV‐1. The second circos illustrates the effect of HSV‐1. The third circos depicts the effect of the SNP allele. The fourth circos shows chromosome density. Red plots represent the *P*‐value < 5 × 10^−7^ and green plots represent *P*‐value < 1 × 10^−5^. The plots were generated using the “CMplot” R script (https://github.com/YinLiLin/R-CMplot). Abbreviations: ADD, additive effect; DOM, dominance deviation.

**Figure 2:**
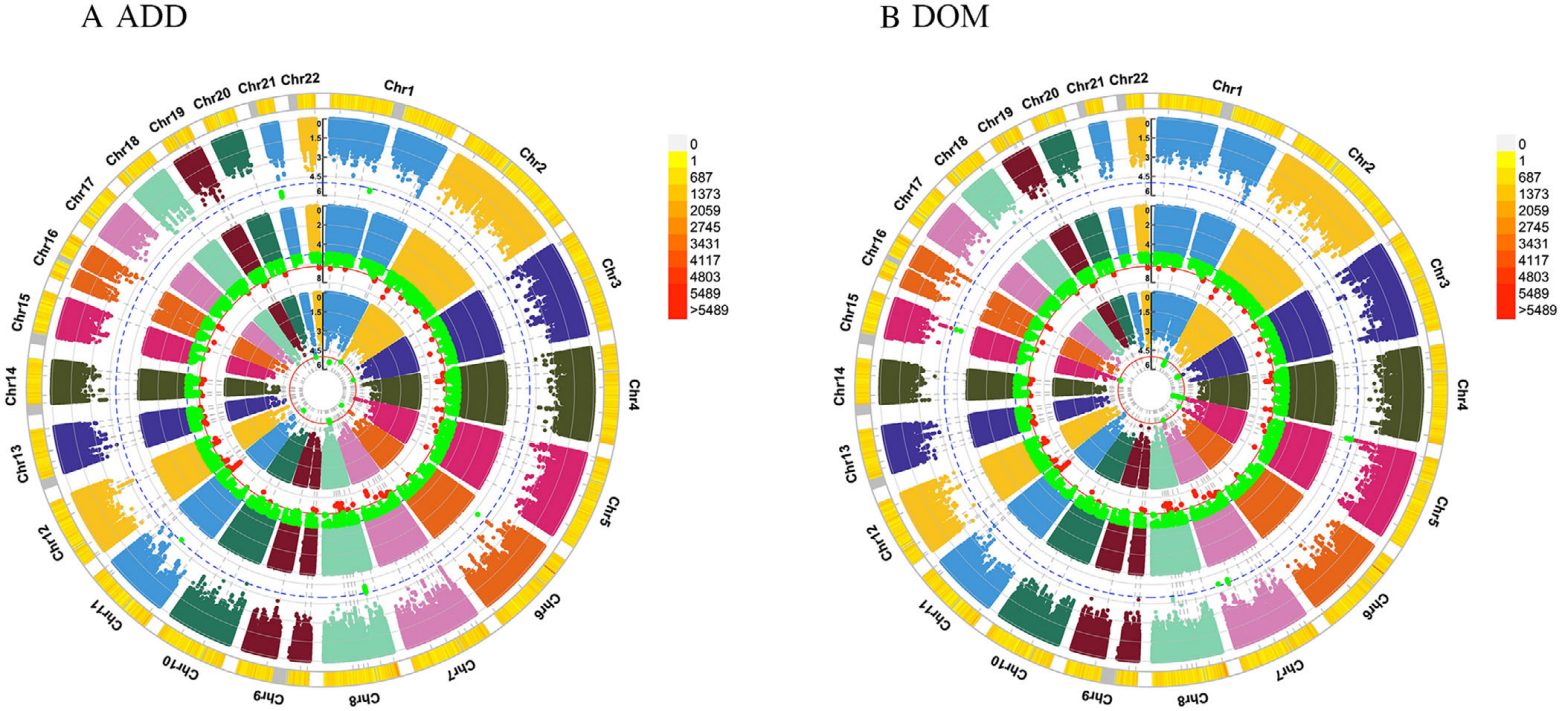
**Genomic regions interacting with HSV‐1 for depression** *Note*. From the center, the first circos depicts the –log_10_ *P*‐values of each variant due to double exposure, that is, the effect of both SNP allele and HSV‐1. The second circos illustrates the effect of HSV‐1. The third circos depicts the effect of the SNP allele. The fourth circos shows chromosome density. Red plots represent the *P*‐value < 5 × 10^−7^ and green plots represent *P*‐value < 1 × 10^−5^. The plots were generated using the “CMplot” R script (https://github.com/YinLiLin/R-CMplot). Abbreviations: ADD, additive effect; DOM, dominance deviation.

For PHQ score, GWEIS identified multiple significant gene × HSV‐1 interactions with *P*‐value < 5.0 × 10^–8^, such as SULF2 (rs6094791, *P *= 8.60 × 10^−9^ for ADD × HSV‐1 model) and TMEM132C (rs11059753, *P *= 1.58 × 10^−8^ for DOM × HSV‐1 model). For depression, suggestive gene × HSV‐1 interactions was detected at ARMC12 (rs113444436, *P *= 1.92 × 10^−6^ for ADD × HSV‐1 model). Additional results are in Tables 3, S1, and S2 and Figures 1 and 2. Subsequently, four significant SNPs for PHQ score were used to perform subgroup analysis between HSV‐1 and depression, including rs9352374, rs9343481, rs11059753, and rs6094791. Significant results were observed in the association between self‐reported depression and HSV‐1 (antibody and seropositivity) in the subgroups. Additional results are in Table S3.

**Table 3:** Summary of gene‐environment interaction analysis between SNP and HSV‐1 for depression and PHQ score

	CHR	SNP	Gene	Model	*P*
PHQ score	20	rs6094791	SULF2	ADD × HSV‐1	8.60 × 10^−9^
	12	rs11059753	TMEM132C	DOM × HSV‐1	1.58 × 10^−8^
	6	rs9352374		ADD × HSV‐1	3.49 × 10^−8^
	6	rs9343481		ADD × HSV‐1	3.49 × 10^−8^
	10	rs1865749		ADD × HSV‐1	7.07 × 10^−8^
	10	rs12358630	ASAH2	ADD × HSV‐1	1.97 × 10^−7^
	10	rs2813305	ASAH2	ADD × HSV‐1	2.08 × 10^−7^
	20	rs6094791	SULF2	DOM × HSV‐1	4.13 × 10^−7^
Depression	5	rs465787		DOM × HSV‐1	1.40 × 10^−6^
	5	rs377094		DOM × HSV‐1	1.87 × 10^−6^
	5	rs414421		DOM × HSV‐1	1.88 × 10^−6^
	6	rs113444436	ARMC12	ADD × HSV‐1	1.92 × 10^−6^
	5	rs160730		DOM × HSV‐1	2.41 × 10^−6^

Abbreviations: ADD, additive effect; CHR, chromosome; DOM, dominance deviation; P, estimates of the effect of interaction on PHQ score by using ADDxHSV‐1 or DOMxHSV‐1; PHQ, Patient Health Questionnaire.

### 3.5 Pathway analysis

**Figure 3:**
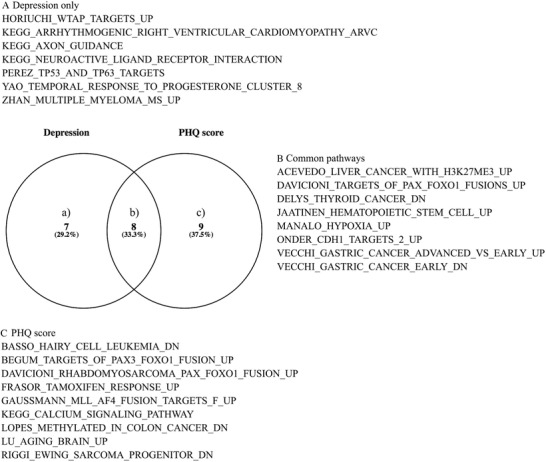
**Distribution of pathways for depression and PHQ score** *Note*. Venn graph shows the distribution of pathways for depression and PHQ score.

Venn diagram showed the distribution of pathways in depression and PHQ score. A total of 15 nonrepetitive significant pathways were identified for depression, such as KEGG_NEUROACTIVE_LIGAND_RECEPTOR_INTERACTION (FDR = 3.18 × 10^−2^ for DOM × HSV‐1 model), YAO_TEMPORAL_RESPONSE_TO_PROGESTERONE_CLUSTER_8 (FDR = 2.32 × 10^−3^ for ADD × HSV‐1 model), and KEGG_AXON_GUIDANCE (FDR = 4.78 × 10^−2^ for DOM × HSV‐1 model). We identified 17 nonrepetitive significant pathways for PHQ score, such as LU_AGING_BRAIN_UP (FDR = 4.21 × 10^−2^ for ADD × HSV‐1 model) and BASSO_HAIRY_CELL_LEUKEMIA_DN (FDR = 3.69 × 10^−2^ for ADD × HSV‐1 model). Additional results are in Table S4. As shown in Figure 3, eight pathways were identified for both depression and PHQ score, seven pathways were identified only in depression region, and nine pathways were identified only in PHQ score region.

## 4 DISCUSSION

Previous studies demonstrated that depression was related to viruses infection that could invade nerves.^25^, ^26^ In this study, we conducted an observational and GWEIS to explore the relationship between HSV‐1 and depression. In UK Biobank cohort, we observed significant association between HSV‐1 and the risk of depression. GWEIS identified multiple gene × HSV‐1 interactions for depression. Our study results suggested that the risk of depression was associated with HSV‐1, which was modulated by the several genes that were related to nerve development or immune function.

Previous studies demonstrated that HSV‐1 was a risk factor for some mental disorders, such as cognitive deficits,^27^ bipolar disorder,^9^ and schizophrenia.^10^ However, there is debate about the association between HSV exposure and depression. For instance, Gale et al demonstrated that HSV‐1 was not associated with an increased risk of depression after adjusted potential confounders, such as age, race, gender, poverty income ratio, smoking status, and alcohol use.^28^ However, in Markkula et al's investigation, the seropositivity and serointensity of HSV‐1 could be associated with the incident of depression after gender classification.^29^ Carter et al's study showed that the susceptibility genes of the HSV‐1/host interactome were significantly enriched in depression.^30^ Additionally, our study results supported that high HSV‐1 antibody or HSV‐1 seropositivity was associated with the increased incidence of depression in UK Biobank. Further researches are needed to draw a definitive conclusion.

To the best of our knowledge, the effort to explore the potential SNPs that influence the association between HSV‐1 and depression was limited. Our GWEIS revealed several candidate genetic variants interacting with HSV‐1 for PHQ score, such as SULF2, TMEM132C, and ASAH2. SULF2, also called HSULF‐2, produces protein heparin sulfatase 6‐O‐endosulfatases, which can selectively remove the 6‐O‐sulfate group from heparin sulfate.^31^ Primary cerebellar granulosa cells isolated from SULF1‐ or SULF2‐deficient neonates were characterized by reduced neurite length and reduced cell survival.^32^ Sulf1/2 double knockout mice showed that Sulfs controlled the formation of corticospinal axons by desulphurization of heparan sulfate.^33^ TMEM132C gene encodes transmembrane proteins of the TMEM132 family proteins. Sanchez‐Pulido et al identified TMEM132 molecules as novel immunoglobulin domain superfamily adhesion molecules containing the central nervous system, connecting the extracellular medium to the intracellular actin cytoskeleton.^34^ Additionally, a family‐based association analyses of imputed genotypes showed a link between TMEM132C and AD, particularly with the age of onset.^35^ ASAH2 produces a ceramidase that catalyzes the hydrolysis of n‐acyl bonds of ceramide (a second messenger), producing a sphingosine that promotes mitosis‐ and apoptosis‐inducing activity, and its phosphorylated form functions as an intra‐ and intercellular second messenger.^36^ Through genome‐wide association analysis of cerebrospinal fluid levels of proteins in 1126 of 133 people, Sasayama et al found the association between ASAH2 and protein levels.^37^

For depression status, although no significant interaction was found, the previously identified gene ARMC12 corresponding to the signal rs113444436 showed a suggestive interaction effect in this study. The proteins encoded by ARMC12 are members of the ARM protein family. The ARM protein family interacts with a wide variety of different binding conjugates through its arm‐repeat domains and performs a variety of basic functions in cell adhesion, intercellular contact, signal transduction, and tumorigenesis.^38^, ^39^ It has been found that blocking the interaction between ARMC12 and RBBP4 by cell‐penetrating inhibitory peptides can activate the expression of downstream genes and inhibit the tumorigenesis and invasiveness of neuroblastoma cells.^40^ Additionally, from phenotype association data using PheGenl and dbGaP data on NCBI website, a lot of immune‐related information was shown to be related with those genes, such as inflammation, immune system, c‐reactive protein, andu so on.

Pathway analysis also provided some evidence for the implication of HSV‐1 in the development of depression through affecting neural development and inflammatory effects. Some identified pathways were related to neural development, such as KEGG_NEUROACTIVE_LIGAND_RECEPTOR_INTERACTION and KEGG_AXON_GUIDANCE. Several identified pathways were related to inflammatory effects, such as BASSO_HAIRY_CELL_LEUKEMIA_DN and YAO_TEMPORAL_RESPONSE_TO_PROGESTERONE_CLUSTER_8. Previous studies found that HSV could infect the central nervous system by moving from the ganglion cell body to the end of the axon,^41^, ^42^ and these effects on nerves and axons might contribute to the development of depression.^43^, ^44^ Additionally, increased inflammation was also associated with depression^6^, ^7^, ^8^ and HSV. Notably, Miller et al pointed out a possible explanation that when certain infections such as neurotrophic pathogens invaded central nervous system, they might activate neuroinflammatory mechanisms and then lead to depression, including HSV, human HIV, and possibly hepatitis C virus.^6^ Therefore, our findings support the previous ideas that the impact of HSV‐1 on depression possibly through SNPs, which are associated with nerve development or immune function.

By reviewing the previous studies,^28^, ^30^ we selected the common confounding factors as covariates in the range of UK data applied. Previous studies have shown that smoking, alcohol use, and the economic status could significantly affect the risk of depression.^45^, ^46^, ^47^ Therefore, we used those factors as covariates to exclude the influence of those factors. Because according to the opinions of previous studies, the number of adjusted confounders with the multivariate adjustment analysis should not be too much in one analysis and should be decided by the number of cases of outcome events,^48^ some other possibly confounders were not considered in this study and need further research. In addition, our population observational study was a cross‐sectional study, so it is not appropriate to make a conclusion about the causality of HSV‐1 and depression solely according to our study results. However, primary infection of HSV‐1 usually occurs early in life (such as in children), leading to the establishment of latent neuronal infections,^49^ which might increase the risk of depression; and we could not exclude the possibility of opposite causality. In addition to the limited genetic studies of HSV‐1 in the previous studies, further cohort studies are expected to draw a definitive conclusion.

Additionally, there are some other limitations in this study. Our analysis results between self‐reported depression and PHQ score were not completely consistent. David et al^15^ demonstrated that self‐reported depression is higher than symptom‐based outcomes, and that there may not be a correct way to categorize all situations. Therefore, choosing multiple measures wisely increases the potential for research. The differences in the study results of self‐reported depression and PHQ score were acceptable and may provide more useful information. Moreover, the significant SNPs from GWEIS are not located in the coding region. Previous studies found most significant SNP identified by genome‐wide association studies located at noncoding chromosomal regions.^50^ These SNP markers are linked to adjacent true causal loci by genetic linkage disequilibrium.^50^ For the SNPs in our study, there is no literature to describe in detail the biological mechanism from SNP status to protein, and further researches are expected.

In summary, we observed significant association between HSV‐1 and the risk of depression using UK Biobank cohort. Further genome‐wide gene‐environment interaction analysis identified multiple candidate genes and pathways, which may contribute to the association. Further studies are warranted to confirm our findings and clarify the potential mechanism of identified gene × HSV‐1 interaction.

## CONFLICT OF INTEREST

The authors declare no conflict of interest.

## Supporting information

Supporting Information

Supporting Information

## Data Availability

The UK Biobank data are available through the UK Biobank Access Management System https://www.ukbiobank.ac.uk/. We will return the derived data fields following UK Biobank policy; in due course, they will be available through the UK Biobank Access Management System.
