# Supplementary material for: Association between herpes simplex virus 1 exposure and the risk of depression in UK Biobank
Source: Clin Transl Med. 2020 Jun 20;10(2):e108. doi: 10.1002/ctm2.108 (PMC7403656; doi:10.1002/ctm2.108)
Supplement: Supplementary file 1 — Supporting Information [file CTM2-10-e108-s001.docx]

Supplement

***Definitions of criterion for depression***

In order to obtain a comprehensive and accurate control group of depressed and non-depressed patients, we self-reported depression, and strictly conducted the control group threshold by Davis et al. research^1^, which based on Patient Health Questionnaire (PHQ-9)^2^ and another strict criterion based on composite international diagnostic interview short-form (CIDI-SF)^2,3^.

Depression phenotype was defined according to three UK Biobank fields: 20002, 20126 and 20544. We selected participants who self reported depression, based on the code 1286 from ID 20002, code 3,4 or 5 from ID 20126 and code 11 from ID 20544 as case.

And for the control of the depression, after excluding the self reported depression defined in our study and depression single episode defined in Davis et al. research^1^,we chose the participants who did not endorse depression or screen positive on PHQ or CIDI. More precisely, participants whose PHQ score ≤5 and did not have core symptoms were selected.

PHQ-9 is a classification algorithm with a total score (0-27) used to screen for and measure depression severity, focusing on nine depressive symptoms and signs (as detailed below：Little interest or pleasure in doing things 20514, Feeling down, depressed, or hopeless 20510, Trouble sleeping 20517, Feeling tired 20519, Poor appetite or overeating 20511, Feeling bad about yourself 20507, Trouble concentrating 20508, Moving or speaking slowly or fidgety or restless 20518, Thoughts that you would be better off dead 20513). In order to meet the 0-3 score for each item of PHQ, the 9 symptom scores (1-4) of our team UK were all reduced by 1 point, which was then added up and participants with PHQ≤5 were selected.

According to CIDI, core symptoms of depression were ID 20446 and ID 20441 in UK Biobank, we chose the participants who response “NO” to the question “Have you ever had a time in your life when you felt sad, blue, or depressed for two weeks or more in a row?” or “Have you ever had a time in your life lasting two weeks or more when you lost interest in most things like hobbies, work, or activities that usually give you pleasure?” as non-depressed patients.

***Definitions of criterion for smoking***

We selected several potential measures of smoking behavior in the UKB. These include: 1) ever-tobacco smoker status; 2) former tobacco smoker status (among ever-tobacco smokers), based on the previous study^6^. We coded ever smoker status as 1 if a respondent reported that they were a current or previous smoker, 0 if they reported never smoking.

***Definitions of criterion for alcohol use***

There are several phenotype options that measure drinking behavior in the UKB. After considering only phenotypes that cover the entire UKB sample, we were left with one: ever alcohol drinking. We coded participant status as 1 if a respondent reported that they were drinking current or previous and 0.

Appendix：Questions of depression in UK Biobank

| Patient Health Questionnaire (PHQ-9) | | | | | | |  |
| --- | --- | --- | --- | --- | --- | --- | --- |
| 1. **20514** 2. **20510** 3. **20534** 4. **20519** 5. **20511** 6. **20507** 7. **20508** 8. **20518** 9. **20513** | Over the last 2 weeks, how often have you been bothered by any of the following problems?  a. Little interest or pleasure in doing things  b. Feeling down, depressed, or hopeless  c. Trouble falling or staying asleep, or  sleeping too much  d. Feeling tired or having little energy  e. Poor appetite or overeating  f. Feeling bad about yourself or that you  are a failure or have let yourself or your  family down  g. Trouble concentrating on things, such  as reading the newspaper or watching  television  h. Moving or speaking so slowly that other  people could have noticed? Or the opposite — being so fidgety or restless that you have been moving around a lot more than usual  i. Thoughts that you would be better off  dead or of hurting yourself in some way | | | [Select one from the following for each of the statements]  - 01 Not at all  - 02 Several days  - 03 More than half the days  - 04 Nearly every day  - DA Prefer not to answer | | |  |
| Core symptoms of depression based on CIDI | | | | | | | |
| **20446** | | Have you ever had a time in your life when you felt sad, blue, or depressed for two weeks or more in a row? | | | | [Select one from]  - 01 Yes  - 00 No  - DA Prefer not to answer | |
| **20441** | | Have you ever had a time in your life lasting two weeks or more when you lost interest in most things like hobbies, work, or activities that usually give you pleasure? | | | | [Select one from]  - 01 Yes  - 00 No  - DA Prefer not to answer | |
| **Smoking：** | | | | |  |  |  |
| **20116** | | | The current/past smoking status of the participant | | [Choose one of]  - 01 Previous  - 02 Current  - 00 Never  - DA Prefer not to answer | | |
| **Alcohol use：** | | | | |  |  |  |
| **20117** | | | The current/past alcohol drinking status of the participant | | [Choose one of]  - 01 Previous  - 02 Current  - 00 Never  - DA Prefer not to answer | | |

1. Davis, K.A.S. *et al.* Indicators of mental disorders in UK Biobank—A comparison of approaches. *International Journal of Methods in Psychiatric Research* **28**, e1796 (2019).

2. Kroenke, K., Spitzer, R.L., Williams, J.B.W. & L?we, B. The Patient Health Questionnaire Somatic, Anxiety, and Depressive Symptom Scales: a systematic review. **32**, 345-359.

3. Kessler, R.C., Andrews, G., Mroczek, D., Ustun, B. & Wittchen, H.l. The World Health Organization Composite International Diagnostic Interview short‐form (CIDI㏒F). *International Journal of Methods in Psychiatric Research* (1998).
